# Foreign Summary

**Published:** 1839-05-25

**Authors:** 


### FOREIGN SUMMARY.

*Clinical Lecture on Eczema Impetiginodes, and remarks on the Contagious and Non-Contagious Pustular Affections of the Head.* By ROBERT CARSWELL.—Gentlemen: Before relating to you the histories of two cases of eczema impetiginodes which have been under your observation, both patients having now left the hospital cured, I shall make a few remarks on the elementary characters of this cutaneous affection, that you may have a more clear conception of those characters, as the only means of enabling you to recognise the disease when you meet with it, in its different forms, and on different parts of the body, and thereby distinguish it from other similar cutaneous diseases with which it is so frequently confounded, and from which it differs in one most essential particular, viz., its non-contagious nature; and this appears to me the more necessary because of the imperfect acquaintance which not only students, but even most medical men, possess of cutaneous diseases generally, and because of the importance of an accurate diagnosis, more especially as regards those pustular forms of cutaneous disease which are propagated by contagion.

In order to impress on your minds the importance of an accurate diagnosis of skin diseases, I may further observe that it is the first and most essential means of acquiring a knowledge of their history and treatment; for as it is, in general, by an accurate appreciation of their *physical* characters that you can obtain their respective designations and names, so is it from this latter circumstance that you can refer to those standard works in which you are to find the result of the experience of those who have studied these diseases in an especial manner. In this point of view alone an accurate diagnosis, if not as important as regards the issue of the case, as it always is in diseases affecting those organs esssential to the maintainance of life, is often much more so as regards the reputation of the practitioner; as, for example, when he pronounces a disease of the skin to be non-contagious which very soon after is communicated to other members of a family, or to the other inmates of a school; or, on the other hand, his pronouncing a disease to be contagious which is not so, and in consequence of his erroneous diagnosis giving rise to great disquietude, and inflicting too frequently a great injury on his patient, as happens to children at school, whose removal follows as a necessary consequence.

These latter observations apply more especially to the pustular and vesiculo-pustular affections of the scalp, some of which are contagious, others not, and which, although in almost all cases their special and distinctive characters are sufficiently well marked to furnish us with the elements of an accurate diagnosis, are frequently, nay daily, confounded with each other.

However frequent the contagious forms of pustular affections of the head are believed to be, it is an important fact that the non-contagious forms are extremely frequent. Perhaps I would be justified in saying that they are much more frequent than the former; for among the considerable number of cases which I have had occasion to treat among the out-patients of this hospital, there have been extremely few of a contagious nature. Indeed, I believe I have had only two cases of contagious pustular disease of the head, viz., the *porrigo scutulata*, more commonly but indefinitely, called ringworm, and certainly not a single case of porrigo favosa.

As I shall, no doubt, have the opportunity of bringing under your notice, at some future period, the subject of pustular diseases of the skin, in their *contagious* forms, I shall not at present enter into a description of their special elementary characters. It will, besides, be sufficient for our present purpose to notice the distinctive characters of these as a means of giving precision and prominency to those which usually characterize the *non-contagious* pustular affections presented by the two patients whose cases I have to relate to you.

And, in the first place, what are the elementary characters of eczema impetiginodes? This disease, as the term implies, is a compound of two diseases,—of eczema and of impetigo. Now, each of these, in its separate state, has its own elementary character—a vesicle in eczema, and a pustule in impetigo. In eczema impetiginodes we have both, the vesicle and the pustule; the vesicle, however, being the primary element, and generally predominating during the early stage of the disease. And, besides, the pustular character of this affection always succeeds to the vesicular, and can easily be traced during its progress to a change in the contents of the vesicle, which, consisting, at first, of a clear yellow-coloured serosity, afterwards becomes milky-looking, opaque, and puriform. In most cases, however, of eczema impetiginodes, the pustular element is much less perfect than the vesicular, the contents of the former consisting of a sero-purulent, rather than of a purulent fluid. But in cases in which the inflammation is more severe than usual, the perfect impetiginous pustule is formed; that is to say, the small, psydraceous pustule, characteristic of impetigo, and even the large or phylaceous pustule, characteristic of ecthyma.

Such are the special and distinctive characters of eczema impetiginodes. The pustular character of this form of eczema distinguishes it from the other forms of the disease, viz., from the eczema simplex, which is a purely vesicular eruption, neither preceded nor accompanied by redness of the skin; and from eczema rubrum, which is always distinguishable by the bright red colour of the skin, and the number of minute visicles by which it is covered. To distinguish eczema impetiginodes from some other diseases of the skin is not always so easily accomplished, and this is more especially the case in that form of scabies, called scabies purulenta, affecting the fingers and hands, parts, also, often affected with eczema impetiginodes. But as these parts were not affected in either of our patients, I shall notice only those circumstances which distinguish this disease more especially from porrigo of the scalp, and on other parts of the body. But I shall first read to you the short case of Charlotte Fuller, admitted on the 1st of January, with eczema impetiginodes. She was a female child, two years of age, in general good health, and about a month before was said to have had *ringworm*, which was followed by an eruption on the head and nates. When examined, the following were the appearances observed:—Scalp thickly covered with an eruption and dried incrustation. In some parts *vesicles*, in others *pustules*, with an *inflamed* basis and a *raised centre*. Behind the ears, erythematous redness accompanied by a considerable discharge. Besides these appearances of the head and ears there was also redness, swelling, and excoriation of the nates. There was little or no disturbance of the general health.

This is an extremely simple and obvious case of two forms of eczema, viz., eczema impetiginodes of the scalp, and of eczema rubrum of the ears and nates. The vesiculo-pustular eruption of the scalp, in the first stage of the disease, and the incrustations formed by the discharge of the secreted fluids in the second stage, were well marked, and without those complications which arise from the long duration of the disease, a bad state of the general health, and neglect of cleanliness. The characters of the eczema rubrum behind the ears and on the nates were less perfect, as the vesicular element was absent, as generally happens on the decline of the disease, there remaining only the bright-red colour of the skin from which it derives its name, with a few thin, laminated, transparent incrustrations, formed by the morbid secretion of the inflamed cutis deprived of the epidermis. In this stage of the affection it resembles, and is sometimes denominated, intertrigo, which, however, is only a variety of erythema, produced by friction of contiguous parts, as between the thighs and nates of fat children, for example.

This case terminated favourably in about three weeks after the admission of the little patient, under the use of a mild antiphlogistic treatment, such as is always indicated and required in recent cases of this nature. After the removal of the hair, poultices were employed with a twofold intention, viz., to facilitate the removal of the incrustations, and diminish the inflammatory excitement which accompanies the eruption. This latter intention was also fulfilled by water-dressings behind the ears. The bowels were regulated, at first, by means of calomel and rhubarb, and afterwards by the compound decoction of aloes and tincture of senna. The local affection improved daily, and the redness and slight discharge that still remained were nearly removed by the application of a lotion of the dilute liquor plumbi, when the child was removed at the desire of her mother.

To make any remarks on the distinctive characters of this case of eczema impetiginodes of the head, and other diseases of this part of the body, would certainly be superfluous as regards the diagnosis of this individual case, so simple and obvious were the elementary characters which it presented. But had this same disease presented itself under more unfavourable circumstances; had the vesicular or vesiculo-pustular character entirely disappeared, and the hair been matted together by the repeated accumulation of the morbid secretion of the inflamed cutis, its real nature might not have been so easily determined. The probability is, that it would have been classed among the porrigos, and suspicions entertained of its contagious nature. And here I may with propriety introduce a few observations on the special and distinctive characters of the contagious forms of pustular affections of the scalp, in order to simplify the means of discriminating between them and other non-contagious pustular eruptions of the impetiginous kind. In the case which I have related no doubt could be entertained, as I have already said, regarding its nature, not only on account of the presence of the vesicles, but from the *form* of the pustule, which the reporter of the case has taken care to state, presented a *raised centre.* This circumstance alone is sufficient to separate the non-contagious from the contagious pustular eruptions of the scalp,—the *form* of the pustule, besides other equally important characters, being the very reverse of the former, viz., having a depressed centre. But, in order to render this subject more precise and intelligible, let me state in outline only, the pustular affections of the scalp. These are four in number: two of them have for their elementary character what is called the favous pustule; the two others, the achores pustule. Now, there can be no doubt that the favous pustule is one *sui generis*, and essentially contagious, and includes two forms of porrigo,—the porrigo favosa, and the porrigo scutulata, the true ringworm of authors, if not of the vulgar. The achores pustules, on the other hand, if they do not characterize a special disease of the scalp, are certainly not susceptible of transmission by contagion, and hence an important distinction between the diseases to which they give rise and those of the favous character. The diseases of the scalp, however, arising in the achores pustule, have been included under the porrigos, and present two varieties, the porrigo larvalis and the porrigo granulata. I am, however, disposed to think, with Biett, that they might be separated from the porrigos, from the circumstance of their non-contagious nature, and also from their bearing a strong resemblance to impetigo or eczema impetiginodes, of which they are probably only modifications, owing to a difference in the seat which they occupy. But, be this as it may, it is obvious that our great object ought to be, to be able to distinguish the contagious pustular eruptions from every other pustular affection of the scalp; and this may be accomplished in by far the greater number of cases either at first sight, or after watching the progress of the disease for a few days. The characters, then, by means of which we distinguish the two forms of contagious pustular diseases of the scalp,—the porrigo favosa and the porrigo scutulata,—are the following; and, first, of those of porrigo favosa: the favous pustule is formed by the deposition of a minute quantity of pus, which concretes almost immediately into a pale yellow or straw-coloured substance, having a defined circular edge, hardly, if at all, rising above the surface of the skin, and surrounded by a slight blush of red. The successive effusion and concretion of the matter proceeds from the centre towards the circumference, in which direction it accumulates, thereby raising the circular edge of the crust, and giving to it that *cup-shaped* form by which it is so readily recognised. The size of these *concrete* pustules varies from one to two lines, to half or three-quarters of an inch in diameter. They are distinct at the commencement, but become confluent during their formation, and are sometimes confounded together into a large, dry, brittle mass, resembling a mixture of sulphur and plaster. Even in this state, however, of agglomeration, traces of the primitive character of the disease are perceptible, viz., numerous round or irregular depressions, indicating the situation and number of the original favi.

In the second form, viz., the porrigo scutulata, the favous pustles, instead of being distinct, as in the former, are confluent from the commencement, and form patches, of various extent, around the circumference of which they are much more numerous than at the centre. Patches of this kind may be seen on various parts of the scalp, but, however much they may increase in extent, by the accumulation of the concrete effused matter, and although, from this circumference, the alveolar depressions may become effaced, the projecting, defined, circular edges of the favi are always to be observed around the circumference of the patches, and serve to point out the nature of the affection. The concrete matter of the patches resembles plaster, is of a dirty gray colour, rather than a yellow tinge, as in the porrigo favosa. Such is a general outline of the physical and distinctive characters of what may be regarded as the true porrigos,—the porrigo favosa and scutulata,—and by means of which we are enabled to distinguish them from other pustular affections of the scalp with which they are confounded, that is to say, with impetigo, eczema impetiginodes, and still more so with the *porrigo larvalis* and *porrigo granulata.* I have already said that these two latter pustular affections have not the favous but the achores pustule for the basis of their classification, and that it is extremely probable that the achores pustule is merely a modification of the pustule of impetigo, affected by its locality, and constitute, when seated in the scalp, varieties of impetigo and eczema impetiginodes. The achores pustules, however, which constitute the porrigo larvalis and granulata, are larger than those of the porrigo favosa and scutulata, at their commencement. They are situated superficially, instead of being sunk deep in the cutis, as is the case in the latter; they are, in fact, prominent, instead of being depressed; are surrounded by an inflamed basis; are scattered over the head or other parts of the body; and in-instead of the puriform fluid *concreting* when effused within a *defined circumscribed* space, it is *spread* over the surrounding surface in the form of laminated, brittle incrustations, of a yellowish-green, yellowish-brown, or brown colour. The dry, irregular incrustations, independently of the colour, of these two *non-contagious* forms of porrigo, cannot be confounded with the solid circular patches, with depressed centres, of porrigo favosa and scutulata, the only two pustular diseases from which, as I have already said, it is of importance that they should be distinguished.

We now come to the consideration of the second case of eczema impetiginodes, which is one of considerable interest, even in a diagnostic point of view, owing to the unusually obscure and complicated appearances which it presented. I shall first read you the history of this case, taken from the case-book, before offering you the explanatory observations which it suggests.

*History of Case.*—John Smith, æt. thirty-five, admitted December 4th, 1838, formerly a groom, but for the last three years has been employed as a gardener; he is of a sanguine temperament, tall and muscular, married, and of regular habits; parents are living, and generally healthy; his own health has always been remarkably good. Fourteen years ago (before his marriage) he contracted gonorrhœa, and got well in about a fortnight by the use of internal remedies. He declares he never had any venereal complaint since, nor, indeed, ever been in "harm's way." In the summer, six years ago, he had an eruption of small pimples all over his body, on glans penis, and scrotum, as well as on other parts. These were attended with very little itching, and died away spontaneously towards winter; they have returned every summer about June. The eruption was supposed, by his medical attendant, to be syphilitic, and the patient was salivated three times within the twelve months, three years ago. At this time he states that he had a small swelling in the groin, which, however, soon subsided after leeching and rest. After the first salivation the eruption assumed a new form; the pimples broke and discharged a yellow fluid, which concreted into thick scabs. Similar pimples now began to appear on the scalp and face, being preceded by severe headaches. Each pimple broke, enlarged, joined with neighbouring ones, and formed large discharging surfaces, which afterwards gradually healed at the centre, on various parts of the head, trunk, and extremities. His throat became sore; there were large ulcers formed in it, and it continued in this state for two months. He became gradually worse and worse, and was, as stated, admitted the 4th December.

*Present symptoms.*—His face is nearly covered with the eruption; the patches are irregular in size, but generally assume a circular form; some parts are erythematous, covered with a furfuraceous desquamation, and around the margins of these patches which have healed in various degrees in the centre, the still discharging eruption forms scabs and crusts of a yellow colour, by the concreting of the matter furnished by the pustules. There are numerous patches on the head, behind the ears, &c. &c.; the margins of the patches are not raised, but the skin around is red and shining; the eruption heals in the centre of the patches, and the parts, once the seat of the disease, do not again become affected. The affected parts are hot, itch, and smart, and heat only makes them worse. There are several large patches on the back, and on the front of the chest, one on the left scapula, and one on the right breast, forming a complete ring. Another very large one is situated just below the knee, healed in the centre, the skin there being of the natural colour, and another patch under the left thigh, four inches in breadth. There are small red papulæ, containing fluid of a yellow colour, like impetiginous pustules, diffused over the body in various parts.

The upper lip is much swollen, and protruded; the eyelids are thickened, there is lippitudo and coryza; the sight is dim and impaired, and the eyeballs bloodshot.

The skin is, at times, very hot and dry; he is very much weakened by the disease; appetite is pretty good; thirst; sleep bad; very little perspiration; bowels regular; urine high coloured, and rather increased in quantity; tongue clean and natural.

The history of this case suggests two inquiries: first, the nature of the eruption considered in itself; and, secondly, its remote cause or origin. As to the eruption, it presented far from common appearances, both as regarded its general characters and the great extent of the surface which it affected; in some of its characters it bore a faint resemblance to psoriasis, particularly in the redness of the inflamed surfaces and the presence of the furfuraceous desquamations, or, rather, thin whitish, transparent, laminated scales, which covered a great part of these surfaces. It was, however, only in these respects that it had any resemblance to psoriasis, and that but a very imperfect one; for, in this disease, the squamæ are white and opaque, and are not only accumulated into thick rugous masses, in chronic cases, such as that of our patient, but the inflamed cutis is thickened, hardened, and fissured, which in this case was smooth and shining. Besides the somewhat scaly or squamous character of the affection, which gave to it a resemblance to psoriasis, there was also another circumstance calculated to lead astray, viz., the tendency of the large patches to heal in the centre; but this circumstance is observed in other and different cutaneous diseases, and particularly in that with which this patient was affected.

Besides these negative characters of the disease, there was one of a positive nature, which at once served to distinguish it from psoriasis, viz., the *incrustations*, or *scabs*, which occupied principally the outer margin or circumference of several of the patches on different parts of the body. These were of a yellowish or yellowish-brown colour, obviously formed by the concretion of a viscid secretion, such, in fact, as is observed to occur in impetigo or eczema impetiginodes. No such kind of crust or viscid discharge occurs in psoriasis although in some cases of psoriasis inveterata, after an exacerbation of the inflammatory excitement, a slight discharge may take place; but even here the resemblance to impetigo is extremely remote in this as well as in many other circumstances.

Could there have been any doubt as to the character of the disease as indicated by the general appearances of the patches, and particularly by that of the scabs, this would have been removed by the presence of the impetiginous pustules on several parts of the body.*

This form of impetigo is, as I have already said, far from being common. It is observed in persons of a lymphatic or scrofulous constitution, and most frequently as a sequela of venereal infection, and possibly in those on whose constitutions mercury exercises an injurious influence. It is stated that this patient had a gonorrhœa fifteen years ago, which was removed in the course of about a fortnight after the use of internal remedies, probably no mercury having been employed. Nine years after, he had, in the summer, what appeared to have been a papular eruption over the whole body, including the glans penis, and which disappeared spontaneously towards the winter, and which had returned every summer since. This eruption was supposed, by the medical attendant of the patient, to be syphilitic, and three years ago he was salivated three times within the twelve months. Instead of this treatment having been of any service to the patient, the disease with which he was afflicted became worse after the *first* salvation. Instead of a papular there now appeared a pustular eruption, occupying first the head and face and accompanied by severe headach. It was at this time, also, that the throat became affected, and was the seat of ulceration for about two months.

From this period, also, the cutaneous disease increased in severity until it had arrived at that stage at which you saw it when he was admitted into this hospital.

When I first saw this patient I did not attach much importance to the venereal origin of his disease, nor was this to me a matter of consequence, as the treatment employed was that which has been found to be, in most cases of this nature, by far the most efficacious.

You have heard that he had fifteen years ago only a gonorrhœa, although our evidence on this point is by no means conclusive. However, were his statement correct, it would not be a solitary instance of syphilitic eruptions succeeding to gonorrhœa after intervals of many years. I have myself witnessed cases of this kind, in which the cutaneous affection itself, either of a scaly, vesicular, pustular, or tubercular character, bore sufficient evidence of its origin; and Biett, of the Hospital of St. Louis, of Paris, who has had the most extensive opportunities of investigating this subject, long since informed me that the occurrence of syphilitic eruptions after gonorrhœa was far from being uncommon.

Numerous experiments, particularly those of M. Ricord, have, indeed, lately demonstrated, that the primary affection of the mucous membrane is, in many cases of gonorrhœa, of the same nature as in chancre, the puriform discharge in these cases, when introduced into the cutis, being followed by the formation of a true venereal sore, or chancre, and its constitutional consequences. From a review of the history of this patient's case, therefore, you will no doubt be disposed to consider the vesiculo-pustular affection which he presented, of syphilitic origin. The sore throat, combined with successive attacks of the cutaneous affection, would, by most physicians, be considered conclusive evidence in a case of this nature.

The treatment in this case was, in the highest degree, successful; how far the cure will be permanent is yet a question. However, the further use of mercury in a case of this kind would have, I am certain, as it already had done, acted most injuriously. Indeed, I may say, that almost all the bad cases of syphilis which I have seen, more especially when the throat was extensively ulcerated, the nose destroyed, nodes of the bones, excruciating pains, &c., have occurred in persons who had undergone repeated courses of mercury, and without imputing this to the deleterious operation of the mercury alone, it is no less an important practical fact that such consequences too frequently follow the operation of this medicine in constitutions contaminated by syphilis.

The following was the treatment adopted in this case:—

Dec. 4. Venesection, ℥xii.; *sol. of hyd. of potash*,* ʒss., thrice a day; middle diet.

6. Blood buffed and cupped; skin less hot.

℞ *Creosote*, one drop;
*Water*, ℥vj.; a lotion for the affected part.

8 Lotion caused some smarting, and was decreased in strength; two ounces additional of water. Increase solution to two scruples.

11. Heat and itching less; eruption paler. *Sol. of hyd. of potass*, ʒi.

15. Improving rapidly. *Sol. of hyd. of potass*, 3½ scruples.

18. Much less redness, heat, and smarting; lotion diminished in strength from its causing too much tingling. *Sol. of hyd. of potass*, four scruples.

22. Eruption still less red and tingling, patient feels much easier, but had an attack of headach and sickness, from having taken ʒss. of the solution, by mistake, more than was ordered.

* A model in wax of a part of the body affected with the disease was exhibited and described.

* The solution of the hyd. of potass employed in the hospital contains one drachm to the ounce of water.

26. Venesection ℥vj.; *sol. of hyd. of potass*, ʒiss.

From this period up to the 10th of January, the general health of the patient and the cutaneous affection gradually and steadily improved.

The use of the creosote lotion was continued, with some variation in its strength, and the solution of the hydriodate of potash gradually increased to ℈v. A few days after the patient was allowed a more generous diet. On the 22nd he was nearly well, desirous of returning home; and on the 24th was discharged cured, the only remains of the cutaneous disease consisting in a reddish discolouration of the parts of the skin which were affected by the eruption.—*Lancet.*

---

*Pituitary Gland.*—Rathke has lately traced the origin of the pituitary gland to a tubular prominence from the membrane of the cavity of the mouth. In many animals, and particularly in mammals, a short time before the formation of the palate, there is seen, far back in the cavity of the mouth, below the base of the skull, an irregular roundish depression in the mucous membrane, but which, at a latter period, is not distinguishable. This is the first stage in the formation of the pituitary gland. As it increases in depth, it passes through the layer of formative tissue at the base of the skull, obliquely upwards and backwards, and, after some time, forms a short blind tube, with a very wide aperture. At a later period there forms, before its entrance, a semilunar fold of mucous membrane, which, as it increases in breadth, covers it like a valve from before backwards. As this takes place, also, the tube narrows at its aperture in the mouth (as the swimming bladder does in its relation to the alimentary canal,) and at last the hole completely closes, and the (now) pituitary gland becomes a perfectly cranial organ.—*Med. Gaz. from Rathke, in Müller's Archiv.* Hift. v. 1838.

---

*John Hunter. From the Medical Portrait Gallery.* By THOMAS J. PETTIGREW, &c.

*Early Life.*—In 1754 he entered as a pupil at St. George's Hospital, of which he became the house-surgeon in 1756, having, in the previous year, been admitted to a participation in the lectures delivered by his brother. A very large portion of the anatomical preparations in Dr. W. Hunter's museum were made by his brother John, and his ability in this matter served to keep the two brothers united much longer than would otherwise have been the case from the dissimilarity of their dispositions and temper. John Hunter was remarkable for his irascibility through life, and it probably served to shorten the duration of his existence. Dr. V. Knox has endeavoured to account for the irritability by which men of genius have so frequently been distinguished. They are, for the most part, he says, in a state of intense thought, while those engaged in matters of less moment, and characterized by less refinement, are often involved in a kind of mental insensibility. As happiness must have its seat in the condition of the mind, it follows that every little accident is likely to disturb the repose of him who is constantly engaged in meditation, as the string which is always kept in a state of tension, will vibrate upon the slightest impulse. "Sensibility of mind and fineness of feelings are always the attendants of true genius." This may somewhat account for Hunter's irascibility.

*As a Teacher.*—As a lecturer Hunter's manner was bad, his language embarrassed, and altogether inelegant; he scarcely raised his eyes from his book, and he much fatigued his class, which was never a numerous one—not exceeding thirty. Hunter was, however, too anxious to display his own views and opinions, and too sensible of the advantages to be derived by the practice of teaching, to give up an engagement, in a pecuniary view, certainly of no consequence. Those only who have lectured can be sensible of the amount of labour required to keep pace with all existing knowledge upon the various subjects for consideration, and the advantage derivable from the necessity of exertion thus created. There is much sound sense in the advice of Erasmus, when he says, "Teach others also; for by no means will you better discover what you understand, and what not."

*A Voice from the Dead.*—Sir E. Home has recorded the particulars of Mr. Hunter's disease and death, many of which were derived from his own dictation.* The account exhibits the history of a complete case of angina pectoris, or that affection which is frequently found to arise from an ossified condition of the coronary arteries of the heart. This had been long foreseen by his friends, and affections of the mind operated powerfully in hurrying on the fatal issue:

> ——— "Disease augmenting year by year,
> Show'd the grim king by gradual steps brought near."

Yet it was sudden—awfully sudden. On the 16th of October, 1793, he went to attend a Board at St. George's Hospital, and there being irritated by some circumstances (the particulars of which may be found in Mr. Ottley's Life of John Hunter, p. 126,) he withdrew from the committee-room into an adjoining apartment, and, turning round towards one of the physicians (Dr. Robertson) he gave a deep groan, and dropt down dead. His existence thus terminated in the sixty-fifth year of his age.

*Temper and Personal Appearance.*—He had no command over his temper, yet he was not wanting in sensibility or benevolence of heart. He was solicitous to advance the fortunes of his most meritorious pupils; he offered to Jenner to accompany Captain Cook upon his voyage of discovery, and he recommended Mr. Thomas as assistant-surgeon to Earl Macartney, embassador to China. His speech was rude, and he habituated himself to the disgusting practice of

* Perhaps Mr. Pettigrew will be kind enough to inform us, in his next number, by what means John Hunter was enabled to *dictate* the particulars of his own death.

swearing, which, in his day, however, was not of uncommon occurrence. He was of temperate habits, particularly during the latter part of life, in which he drank no wine. This was, probably, necessary from attacks of gout to which he was liable. In the acceptance of fees from his patients he was very considerate, particularly towards professional men of all descriptions, and he has been known to return fees, where he found his patients in embarrassed circumstances. As an operator, he has been variously described. In the performance of these painful parts of a surgeon's duty he appears to have been "slow and sure." His knowledge of anatomy would necessarily give security in these undertakings. His personal appearance has been thus described:—

"He was about the middle stature, of a vigorous and robust frame, and free from corpulency; his shoulders were high, and his neck short. His features were rather large, and strongly marked; his eyebrows projecting, his eyes of a light colour, his cheeks high, and his mouth somewhat underhung. In dress, he was plain and gentlemanlike; and his hair, which in youth was of a reddish-yellow, and in his latter years white, he wore curled behind."

*Museum.*—The Hunterian museum has been frequently described. Catalogues are in the course of publication by the Royal College of Surgeons, of its various parts, by Mr. Clift and Professor Owen, under the guidance of the trustees and the Board of Curators, and will form an exceedingly valuable contribution to anatomical, physiological, and medical science, as well as to natural history in general. It is sufficient here to state, that the museum was purchased by the Government, of Mr. Hunter's family, for the sum of £15,000, (though Mr. H., as he told Mr. Lynn, expended no less than £70,000 upon it,) and was presented to the Royal College of Surgeons upon certain conditions, namely, to preserve the collection at their own expense—to have it open to the Fellows of the Royal College of Physicians, to the members of the Royal College of Surgeons, and to persons properly introduced; the preparation of a proper catalogue; officers to explain the collection; and an annual course of twenty-four lectures on comparative anatomy, to be illustrated by the collection. In 1806 a grant by Government of £15,000 was made, to assist in the erection of a proper building for the museum, theatre, &c., and, in 1810, a further sum of £12,500.

---

*Clinical Remarks on Amputation—Excision of Joints.* By Mr. Liston.—Mr. Liston said that, at the commencement of the session, he had given several clinical lectures on the diseases of joints; he had then an opportunity of amply illustrating his remarks by cases in the hospital. He had, in those lectures, strongly recommended to the students the attempt to save the upper extremity by the excision of the joints; but, at the same time, impressed upon them the necessity of its limitation to certain cases. Two cases had lately occurred in which, had circumstances allowed, it would have been most desirable to have saved the extremity by cutting out the elbow-joint. One of these cases was in the hospital; the child was only three years of age, and it was the right arm that was affected. On carefully examining the diseased structures, however, he determined on the performance of amputation. The child's health was so delicate that the severe operation of excision would, probably, have proved fatal, by the shock produced by it upon the system; and the local mischief was so extensive as to have rendered it impossible to have been entirely removed by excising the joint; indeed, as they would perceive by the specimen, the joint itself was, comparatively, but little affected, the only mark of disease being at the junction of the epiphysis with the shaft of the ulna, where the bone was exposed, by ulceration, to a very slight extent. The chief disease was situated in the lower end of the shaft of the humerus; there was necrosis of the cancellated structure, and a portion was on the point of exfoliation. Had excision been attempted in this case, the removal of about one-third of the affected bone would have been rendered necessary; and with such a proceeding the termination would most probably have been unfavourable. The child had suffered from superficial abscesses, and had become hectic; but a great improvement had taken place in the general health, and the local affections might also be expected to disappear, now that the principal hectic cause had been removed. In another case, in his private practice, that of a child about the same age as the former patient, it would have been equally desirable to have saved the arm by excision of the elbow-joint, had such a proceeding been practicable. But in this instance, as in the last, the disease was of such a character, and so situated, as to render amputation the only available means of treatment. As they would perceive, besides extensive destruction of the articulation, the shaft of the ulna was much diseased, an abscess having formed in that bone, and there was thickening of it from the elbow nearly to the wrist-joint. Nothing less than the removal of the entire ulna would have been effective. The arm was amputated, and the child has done well.

The pupils would see from these two cases the absolute necessity of carefully examining into the actual condition of a part before they attempted any operative procedure. From the neglect, moreover, of strict inquiry into the previous history of cases, much mischief had resulted. Thus, in many instances, the products of disease had been mistaken for the results of recent injury, and steps resorted to which rendered a previously mild disease one of a formidable character.

In one of the lectures given in the early part of the session, on the diseases of joints, he had related an instance in which an incipient morbus coxarius had been mistaken for a dislocation, and means resorted to for its reduction. A careful inquiry into the previous history of this case would have prevented the occurrence of the mistake, the child having walked about for many days after the receipt of the trifling accident which was supposed to have caused the displacement. He also referred to a case in which a surgeon performed the operation of excision of the elbow-joint, in a child whose ulna was afterwards found to be diseased to a great extent: the termination, of course, was anything but favourable.—*Lancet.*

---

*On Prolapsus Recti.* By Mr. Liston.—The pupils had seen him frequently operate, of late, for prolapsus recti, and he had again and again pointed out the absurdity of removing, by ligature or otherwise, any portion of the lining membrane of the bowel. When hæmorrhoids first formed, the coats of the veins were but slightly thickened, the tumours being soft; they then became hard, solid, and condylomatous, from the coagulation of their contents and consequent changes. The bowel, from the constant straining, protruded, often to a great extent, both when the patient was at stool and at other times. The protruded bowel furnished a profuse mucous discharge, and the veins ramifying under the mucous lining of the bowel, their circulation being interfered with by the pressure of the sphincter, often gave way, by which much blood was lost; it became necessary to put a stop to these discharges. Now, it had formerly been much the fashion to remove, by incision, the folds of the mucous membrane, which are denominated internal piles. It had been found, however, by Sir A. Cooper and Dupuytren, that this proceeding was not free from danger, some patients under these distinguished surgeons having actually lost their lives from hæmorrhage, whilst others had made very narrow escapes after this plan had been followed. It was also customary, as they might be aware, to strangulate portions of the bowel which protruded, by passing ligatures through them. These were also, occasionally, transfixed by long pins; this proceeding was attended by great suffering, and was not free from danger, and appeared to be totally unnecessary. All that was required to be done was to pinch up a portion of the loose integument and altered bowel, changed in character from being constantly protruded, and excise it in the axis of the bowel, either by a knife or scissors. By this plan the action of the sphincter was no longer interfered with, and the contraction of the parts, by cicatrization and adhesion to the subjacent tissues, effectually prevented the protrusion of the bowel.—*Ibid.*

---

*On Cirsocele.* By Mr. Liston.—A patient had been in the hospital with cirsocele, affecting both sides of the scrotum. This disease, Mr. Liston observed, though not a dangerous, was a very common and troublesome one. The scrotum, in some instances, was so much relaxed as to reach about half way down the thigh. So inconvenient was this affection occasionally, that he had been induced, many years since, at the earnest solicitations of the patients, to remove the testicle of the affected side in two cases.

In one of these cases the man was a saddler, and some years before had entered the Royal Infirmary of Edinburgh for hydrocele, which was treated by injection, but the disease returned, and on the second occasion it was treated by incising the scrotum, Mr. Bell being the operator. Some years after this he again presented himself to Mr. Liston, labouring under cirsocele. In consequence of the testicle getting constantly between his thigh and the clams, (a machine used in their work by saddlers,) he requested that the testicle might be removed. Castration was accordingly performed, and the man did well. The second case was that of a farmer, who, being in the habit of taking much horse exercise, found great inconvenience from the pressure of the testicle against the saddle, which could not be prevented by any contrivance in the way of bandage which he had employed; he had suffered from cirsocele for many years. The testicle was removed. He (Mr. Liston) would not now, probably, be induced to perform such an operation; he mentioned the cases to show how serious was the inconvenience which occasionally arose from the disease under consideration.

Cirsocele was a disease usually affecting the left side of the scrotum, which was accounted for partly by the mode in which the spermatic vein entered the emulgent on this side, by this testicle hanging lower than that on the right side, and by the pressure exerted by the sigmoid flexure of the colon. The disease was rarely found on the right side, and more rarely still did it, as in the case under consideration, affect both sides. Occasionally the disease attained a very large size, and might, by a careless observer, be at first mistaken for hernia. He had seen many instances in which instruments had been applied to cirsoceles, under the suspicion that they were cases of hernia. The instruments, of course, would only aggravate the disease; the diagnosis, however, between cirsocele and hernia, would be found in all the elementary works. In cirsocele the tumour, as in hernia, disappears when the patient is in the recumbent position; it subsides under pressure, and an impulse is conveyed to it on coughing; but if the tumour was reduced, and you applied pressure against the external aperture, the swelling would again take place, whether the patient was standing or lying.

In the generality of cases of cirsocele, the palliative treatment, by bandages to suspend the relaxed part, and the application of cold and astringents, would prove sufficient. In addition to these, Mr. Wormald had also proposed a plan for diminishing the capacity of the scrotum, and by that means controlling the disease. To effect this purpose, he drew a portion of the scrotum through a metal ring. When patients became fidgetty, or the disease was assuming a more serious character, then it became necessary to resort to other methods of treatment. Many plans had been devised and practised for the cure of cirsocele. Castration had been performed by many surgeons. Celsus had recommended that a heated needle should be passed into the spermatic veins, and that they should be thus cauterized. He (Mr. Liston) had adopted this plan in a number of cases, both in hospital and private practice, but the remedy, though effectual and safe, was a severe one. It had been proposed, also, to divide the skin and cut out the enlarged veins; but this practice, from its results in the hands of Sir Everard Home, was not encouraging. Mr. Mayo had recommended and practised the application of caustic potash to the spermatic veins in the same way that he had applied it to the veins in the lower extremity. You could not, however, control the action of this agent, which might spread farther than was intended, and destroy the vas deferens and the nerves and vessels going to the gland. Dr. Fricke, of Hamburgh, had advised the passage of a seton through the affected veins. Another mode had lately been followed by Sir A. Cooper, and this consisted in the removal of a large portion of the scrotum. Sir Astley considered the scrotum would, by the contraction resulting, be able better to support the testicles, and the veins would be also thus compressed. Four cases of cirsocele had been treated in this way, and published in "Guy's Hospital Reports;" three by Sir A. Cooper, and one by Mr. B. Cooper. In one case, a piece of the scrotum measuring four inches by two and a half, was removed. In Mr. B. Cooper's case, recorded in the last number of the "Reports," violent inflammation of all the surrounding parts followed the operation, and Mr. Cooper attributed the success of the proceeding more to the effects of the inflammation than to the removal of the scrotum. He says that "the inflammation produced obliteration of the veins, and effected a cure." The operation was, however, a very severe one, and other means of obliterating the veins might be more safely as well as effectually employed. In Sir A. Cooper's plan there was much immediate risk, and if the edges of the scrotum, after division, were to be brought together, there would be much fear of the extravasation of blood and putrid serosity into the cellular membrane.

In addition to these objections, the patient was liable to be confined to his bed for a long period; thus it appeared that one operation was performed on the 8th of February, and the patient could only sit up a little on the 21st of the same month. The practice followed in the case under his (Mr. Liston's) care, was one which had been recommended by M. Davat, and the pupils had seen him, Mr. L., adopt the same proceeding in varicosed veins of the lower extremity. The plan, as modified, and he, Mr. L., thought improved upon, consisted in the passage of two or three needles, at the distance of half an inch from each other, under the enlarged bunches of veins, taking care to avoid the other parts of the chord. Ligatures were then passed over the extremities of the needles so as to employ pressure upon the veins. The needles were taken out at the end of three or four days, when the contents of the veins had coagulated, and a cure was effected. The veins were in this way diminished in size, the weight of the column of blood being removed, and the functions of the testicles preserved entire. The pupils had seen this practice followed in a variety of cases of enlarged veins, and no bad results followed in any instance. He always removed the needles early, before suppuration had set in, or any process for the discharge of the foreign body had made any progress.—*Ibid.*

---

*Case of Hæmorrhagic Idiosyncrasy, with Remarks.* By Mr. Liston.—A man named S. P., had been just discharged cured from the hospital; his case was one of some interest. He is a farmer, thirty-two years of age, and temperate in his habits. He was admitted March 8th, 1839. He is of a hæmorrhagic diathesis, and he states that this is hereditary; that his maternal grandfather was of the same idiosyncrasy, and bled to death from a spontaneous hæmorrhage taking place from the nose and mouth, after having been affected with fever. He does not know whether his grandfather had any relations similarly affected. His mother is a little, delicate, fair-complexioned woman, but not of a similar diathesis. He has six brothers, all with the same peculiarity of constitution. None of his five sisters (although three from the ages of nineteen to to twenty-five are very unhealthy) are affected with it. Four of his brothers, as well as the patient himself, have lost alarming quantities of blood, at different times, from the extraction of teeth. The hæmorrhage was generally, at length, commanded by pressure, though, in one instance, the actual cautery was applied. The case of one of them is recorded by Mr. Kendrick, of Manchester-street, in the 5th vol. of the "Medical Gazette," page 788. The bleeding from the socket of a tooth was so profuse, and returned so repeatedly, that the patient was reduced to the lowest ebb. Mr. Kendrick at last succeeded in arresting the bleeding by the application of pledgets of cotton wool soaked in the strongest spirit. The ligature of the carotid, even, was contemplated in this case.

Several of his brothers have received wounds in various ways, from which, however slight, they have always experienced great difficulty in suppressing the hæmorrhage.

The patient himself is a large, strong made man, with a smooth, fair complexion, dark gray eyes, dark hair, sandy whiskers, and pulse full, about eighty-four. When about nineteen years of age he had a small wart cut from his hip, from which he lost so much blood that he fainted, and for some days after experienced great debility. He has, too, as stated, suffered from the extraction of teeth. Some weeks ago, he says, he had a fall, and hurt his back and loins. Some days after a pain came suddenly in the left groin, accompanied also with a pain in the thigh shooting downwards to the knee. Upon examination he found a swelling in the groin, upon which he took the advice of a surgeon, who ordered leeches and fomentations. Only two leeches were applied, the hæmorrhage from which could not be suppressed till the occurrence of syncope. There is now a collection of matter in the left groin, about the size of the patient's fist. Such, then, was the history of this case; the tumour itself was interesting, it was not in the usual situation of bubo, but lay directly over the inguinal canal, and might have been mistaken for hernia. The history of the case, however, would have shown that it could not be a hernial swelling, coming on gradually, as it did, after a recent injury. The hæmorrhagic diathesis of the patient was also interesting; he, Mr. Liston, had seen cases similar in many respects. He recollected the case of an old gentleman, in tolerable health, from whom he removed a very small tumour from the forehead,—a kind of warty excrescence not larger than the end of the finger,—considerable hæmorrhage took place. The aqua styptica, a solution of alum and the sulphate of copper, caustic, pressure, and even the application of the actual cautery, failed to arrest the bleeding. At length, by having kept up pressure with the finger upon a piece of cork for several hours, the hæmorrhage ceased. This patient's nephew, who accompanied him, had informed him, Mr. Liston, that his whole family were of a similar diathesis, and that one of them had nearly died from hæmorrhage consequent upon the extraction of a tooth. Mr. Blagden had related a case in the 8th volume of the "Medico-Chirurgical Transactions," of a man who perished from the bleeding following the removal of a tooth, although the use of the actual cautery, and every kind of local application, had been resorted to, and the common carotid tied. Some years before, the extraction of a tooth had nearly proved fatal from the same cause. In examining the vessel, they found the coats very thin and transparent; "several opaque, white depositions" were noticed on "the outer surface of the inner coat, such as precede ossification." He, (Mr. Liston,) however, thought that it was equally necessary in such cases to examine into the state of the blood, as well as the condition of the vessels, as much, no doubt, depended upon that. In the case of S. P., he was fearful of using the knife, and therefore opened the abscess with the potassa fusa, and hence there was but slight bleeding. The blood, (which had been examined through a microscope,) although the man looked so strong and healthy, differed from the usual character of this fluid, and seemed to be deficient of fibrine; the globules were broken down, being as might be called "diffluent." The blood was altogether in a bad state, containing a proportion of globules with all the characters of those entering into the composition of pus. These were sometimes observable in the blood of persons apparently not much out of health. They were to be found uniformly in the blood of patients labouring under local disease with much constitutional disturbance, even before the appearance of suppuration in the cellular tissue or upon the surface. In patients labouring under the effects of severe injury, or wasting discharge, internal or external, pus globules could readily be detected. In the blood of that patient in ward No. 2, labouring under the effects of compound fracture of the thigh, with diseased knee-joint, and in that of the patient who had, on account of secondary hæmorrhage, had first the femoral and then the external iliac tied, they had already had an opportunity of examining them. It was gratifying to find, however, that in this latter case an immense improvement had taken place in the quality of the circulating fluid corresponding with his amended state of health. The blood was thicker, the globules were now quite distinct and defined in their edges, and the presence of pus globules could no longer be discovered. The alteration in the qualities of the circulating fluid, in some cases, was apparent enough, even to superficial observers. It might, in those who had been exhausted by disease, by profuse discharges, by repeated loss of blood, who had been badly nourished, or had suffered from depressing passions, fear, and anxiety, be noticed to be thin and watery, to coagulate slowly, and to be very deficient in colouring matter. The blood of some patients who had been under treatment lately, as that little boy, who suffered amputation of the arm, alluded to in the commencement of these observations, also in another patient who had repeated bleeding from a stump of the thigh, and in whom the femoral was tied, had undergone most palpable changes. It was more like the water in which one's hands and instruments had been washed after an operation, than healthy blood; and from its want of coagulability, it oozed out in abundance from every breach of surface. Hence the difficulty of arresting hæmorrhage in such states of constitution. S. P. was dismissed cured on the 2d inst.—*Ibid.*

---

*Statistics of St. Petersburgh.*—The *Abeille du Nord* gives the report of the Prefect of Police of St. Petersburgh for the year 1838. It appears from this document that the population of the capital is 469,720, among which are only 136,000 women. The number of births was 10,437, and the deaths were 7,275. During the whole year there were only seven murders and thirty-four suicides.

In the month of August, the small-pox ravaged several quarters of the town, and attacked persons who had been vaccinated. In other respects the sanitory state of St. Petersburgh was very satisfactory.—*Gazette des Hôpitaux.*

---

*Blood-Globules of the Camel and Alpacha.*—At the *séance* of the institute of December 30th, MM. Milne Edwards and Isidore St. Hilaire presented their report on the investigations of M. Mandl, on the structure of the blood-globules of various animals. They confirmed his assertion, that of all that he had examined (including many of the rarer mammalia,) those of the dromedary and alpacha alone presented any other than the circular form; and that these were elliptical, their diameters being in the proportion of 1-235 to 1-230.—*Lon. Med. Gaz.*

